# Molecular biomarkers for facilitating genome‑directed precision medicine in gynecological cancer (Review)

**DOI:** 10.3892/ol.2023.14012

**Published:** 2023-08-17

**Authors:** Takeo Minaguchi, Ayumi Shikama, Azusa Akiyama, Toyomi Satoh

**Affiliations:** Department of Obstetrics and Gynecology, Institute of Medicine, University of Tsukuba, Tsukuba, Ibaraki 305-8575, Japan

**Keywords:** biomarker, gynecological cancer, companion diagnostic, PARP inhibitor, immune checkpoint inhibitor

## Abstract

Prominent recent advancements in cancer treatment include the development and clinical application of next-generation sequencing (NGS) technologies, alongside a diverse array of novel molecular targeting therapeutics. NGS has enabled the high-speed and low-cost sequencing of whole genomes in individual patients, which has opened the era of genome-based precision medicine. The development of numerous molecular targeting agents, including anti-VEGF antibodies, poly (ADP-ribose) polymerase inhibitors and immune checkpoint inhibitors, have all improved the efficacy of systemic cancer therapy. Accumulating bench and translational research evidence has led to identification of various cancer-related biomarker profiles. In particular, companion diagnostics have been developed for some of these biomarkers, which can be clinically applied and are now widely used for guiding cancer therapies. Selecting biomarkers accurately will improve therapeutic efficacy, avoid overtreatment, enable earlier diagnosis and reduce the cost of preventing and treating gynecological cancer. Therefore, biomarkers are fast becoming indispensable tools in the practice of genome-directed precision medicine. In the present review, the current evidence of cancer-related biomarkers in the field of gynecological oncology, their molecular interpretations and future perspectives are outlined. The aim of the present review is to provide potentially useful information for the formulation of clinical trials.

## 1. Introduction

Notable advancements that have been encountered in the field of cancer treatment over the preceding decades include the development of next-generation sequencing (NGS) technologies and the clinical application of various molecularly-targeting therapeutics. NGS has enabled the high-speed and low-cost sequencing of whole genomes in individual patients, which opened the era of genome-based precision medicine (1). In addition, the development of numerous molecular targeting agents, such as anti-VEGF antibodies, multikinase inhibitors, poly (ADP-ribose) polymerase (PARP) inhibitors and immune checkpoint blockers has improved the efficacy of systemic cancer therapy by combining with conventional chemotherapeutics and with different types of targeting agents (2). This improvement in efficacy has even been observed for patients with advanced or metastatic tumors with inflamed phenotypes (2). Accumulating research evidence has led to the detailed elucidation of a number of oncogenic signaling pathways, e.g. cell cycle regulation, apoptotic signaling, kinase signaling, DNA damage response, DNA mismatch repair, and immune checkpoint signaling, resulting in the identification of a variety of cancer-associated biomarkers. Cancer-associated biomarkers can in turn be utilized for a number of specific purposes, including predicting patient prognosis, predicting tumor response to chemotherapeutic and molecular targeting agents, early diagnosis or prevention of cancer and aiding precise diagnoses. In addition, companion diagnostics have been developed using some of the biomarker profiles, which can be applied clinically and are now becoming widely utilized for guiding cancer treatment (3). These include BRACAnalysis (Myriad Genetics), myChoice (Myriad Genetics) and FoundationOne CDx (Foundation Medicine) (3). Selecting the appropriate biomarkers are predicted to improve treatment efficacy, avoid overtreatment and reduce the cost of preventing and treating gynecological malignancies, rendering biomarkers essential tools in precision medicine. In the present review, current evidence of cancer-related biomarkers in the gynecological oncology field was summarized. This will be based on findings extracted from clinical trials, their molecular interpretations and future perspectives.

## 2. Biomarkers for predicting patient prognosis

Biomarkers used for predicting prognosis prior to commencing treatment are expected to be beneficial for stratifying patients based on the recurrence risks, to designate the optimal combination of treatment modalities and therapeutics.

### Endometrial cancer (EC)

ECs were conventionally classified into two groups, type I and II, based on clinicopathological, epidemiological and endocrinological features (4). Type I EC is characterized by low-grade endometrioid histology and tend to more frequently develop in younger and obese women. In addition, the pathogenesis of this type of EC is associated with unopposed estrogen, superficial myometrial invasion, early stage at diagnosis and favorable prognosis. By contrast, type II EC is characterized by high-grade, non-endometrioid histology, tend to occur in older and more slender-shaped women, deep myometrial invasion, advanced stages and poorer prognosis. Commonly mutated genes in type I tumors include *PTEN, PI3K* catalytic subunit α, *KRAS*, AT-rich interactive domain-containing protein 1A and β-catenin 1, whilst *TP53* is more frequently mutated in type II tumors (5). This dualistic classification system has been pivotal for understanding this entire disease entity in terms of EC pathogenesis. However, this categorizing model is considered incomplete, due to intergroup overlapping caused by tumor diversity and heterogeneity (5).

In 2013, molecular analyses of 232 endometrial carcinomas by The Cancer Genome Atlas (TCGA) program on the results of exome sequencing, microsatellite instability (MSI) testing and microarray of somatic copy number alterations resulted in the proposal of classifying EC into four genomic categories. They are *POLE*-ultramutated [1], microsatellite instability hypermutated [2], copy-number low [3] and copy-number high [4], each showing distinct progression-free survival (PFS) profiles (6). Of note, POLE is a catalytic subunit of DNA polymerase ε, which is involved in nuclear DNA replication and repair.

**Figure 1.**
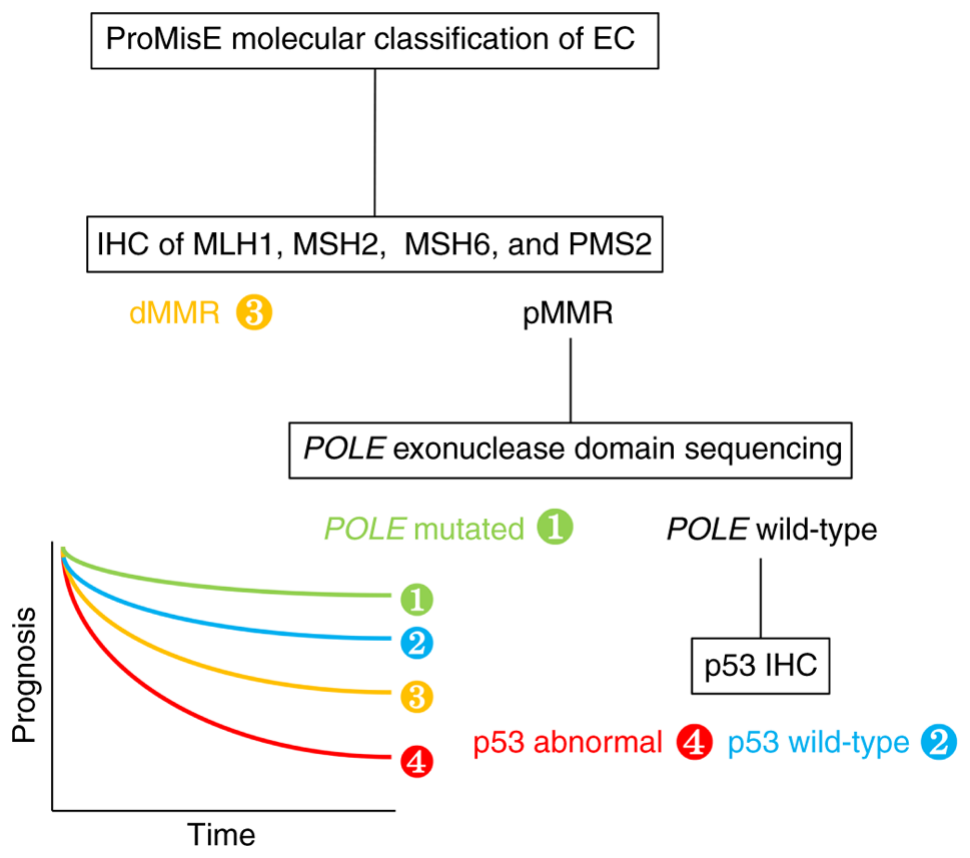
ProMisE molecular classification of EC (7). dMMR, *POLE*-mutated, p53 abnormal and p53 wild-type tumors are identified using the respective molecular methods. *POLE*-mutated tumors show the most favorable prognosis, followed by p53 wild-type, dMMR and p53 abnormal tumors showing the worst prognosis. EC, endometrial cancer; IHC, immunohistochemistry; dMMR, deficient mismatch repair; pMMR, proficient mismatch repair; POLE, DNA polymerase ε.

In 2015, a more clinically applicable, cost-effective classification system, named ‘ProMisE’, was developed using the same TCGA data and applied it to a new cohort of cases (7). The four categories of *POLE*-mutated [*POLE* exonuclease domain mutation (EDM) was determined by sequencing] [1], p53 wild-type [2], DNA mismatch repair deficiency [determined by immunohistochemistry (IHC) testing of MutL homolog 1 (MLH1), MutS homolog (MSH)2, MSH6 and postmeiotic segregation increased 2 (PMS2)] [3] and p53 abnormal (p53 status was determined by p53 IHC) [4] revealed significantly different overall (OS; P=0.0082, n=141), disease-specific (P=0.0378, n=139) and recurrence-free survival (RFS; P=0.0358; n=133; Fig. 1).

In 2017, the ProMisE molecular classification system was confirmed in a large cohort of 319 EC samples, showing distinct OS (P<0.0001), disease-specific (P<0.0001) and PFS (P<0.0001) (8). In particular, the *POLE* EDM group had the most favorable outcome, whereas the p53 abnormal group had the worst outcome. To conclude, using these genome-based risk factors to stratify the patients into the various adjuvant therapeutic regimens may improve treatment outcome and reduce overtreatment. However, prospective studies, such as PORTEC-4a (Molecular Profile-based Versus Standard Adjuvant Radiotherapy in Endometrial Cancer; NCT03469674) and TAPER (Tailored Adjuvant Therapy in *POLE*-mutated and p53-wildtype Early Stage Endometrial Cancer; NCT04705649), are currently on-going, from which additional results are expected (9,10).

## 3. Biomarkers for predicting tumor response to chemo-therapeutic and molecular targeting agents

Biomarkers used for predicting the efficacy of systemic therapeutics are expected to be useful, especially for advanced or aggressive tumors, where localized therapies alone are insufficient.

### Ovarian cancer

The majority of ovarian cancer cases are diagnosed already at advanced stages and chemosensitivity is one of its most important prognostic factors. NGS was previously performed on 393 patients with ovarian carcinoma who received primary surgery. They were also prospectively followed-up for survival analysis. Multivariate logistic regression on 281 high-grade serous carcinoma (HGSC) cases adjusted for germline or somatic *BRCA* mutations, age at diagnosis, optimal cytoreduction and neoadjuvant chemotherapy was performed. It was found that the presence of any *TP53* mutations were associated with platinum sensitivity, which was defined as time to progression after the completion of adjuvant platinum chemotherapy (odds ratio, 0.41; 95% confidence interval, 0.17–0.99; P=0.048) (11). This observation suggests that the presence of *TP53* mutations may predict platinum sensitivity in patients with HGSC.

The PARP inhibitor olaparib was previously evaluated in a phase I trial in 60 refractory solid tumors (12). An expansion cohort of this trial was studied further in patients with ovarian cancer harboring germline breast cancer gene (*BRCA*)1/2 mutations, revealing significant associations between platinum-free interval and the maximal % tumor response rate after olaparib treatment (radiological change, P=0.001, n=38; cancer antigen 125 change, P=0.002, n=46) (13). This finding suggests that platinum sensitivity of patients with germline *BRCA1/2* mutations may predict the response to PARP inhibitors.

The efficacy of maintenance olaparib therapy was also evaluated by the PAOLA-1 phase 3 trial, which compared olaparib treatment with placebo in patients with newly diagnosed stage III/IV ovarian cancer who showed a response to first-line platinum-taxane plus bevacizumab followed by maintenance bevacizumab (14). The hazard ratio (HR; 95% CI) for disease progression or death for olaparib vs. placebo was 0.33 (0.25–0.45) in homologous-recombination deficiency (HRD)-positive tumors and 1.00 (0.75–1.35) in HRD-negative tumors. Tumor HRD status was determined by Miriad myChoice CDx testing, which is designed based on germline/somatic *BRCA1/2* mutations and/or positive genomic instability scores using DNA isolated from formalin-fixed paraffin-embedded (FFPE) tumor tissues. This finding suggests that HRD positivity can be used to predict the efficacy of adding maintenance PARP inhibitors into the primary treatment strategy for advanced ovarian cancer.

The safety and activity of niraparib monotherapy was evaluated by the QUADRA phase 2 trial in patients with relapsed high-grade serous ovarian cancers treated with ≥3 chemotherapy regimens (15). In patients whose tumors were platinum-sensitive to the most recent line of platinum therapy (n=105), the overall response rate was 26% in HRD-positive tumors compared with 4% in HRD-negative or unknown tumors. HRD status was also determined using the Miriad myChoice CDx testing. This finding suggests that HRD positivity can also be applied to predict the effectiveness of PARP inhibitor monotherapy for patients with recurrent platinum-sensitive serous ovarian cancer.

### EC

The PORTEC-3 is a phase III trial that investigated the benefit of chemoradiotherapy compared with radiotherapy alone for high-risk endometrial cancer (endometrioid G3 stage IA with lymphovascular space invasion; endometrioid G3 stage IB; endometrioid stage II–III; and non-endometrioid stage I–III) (16). Using tissue samples from this trial, the prognostic value of a molecular classification system similar to ProMisE (7) was evaluated. Significant improvement in the RFS was found with the addition of adjuvant chemotherapy to radiotherapy for p53 abnormal tumors (5-year RFS, 59 vs. 36%; P=0.019; n=93) (17). p53 status was evaluated by IHC and if applicable, the *TP53* mutational status was also analyzed by NGS. This finding suggests that the p53 mutational status can be used to predict tumor chemosensitivity or enhancing effect of radiosensitivity by chemotherapy in patients with high-risk EC.

KEYNOTE-028 is a phase Ib trial in patients with programmed death ligand 1 (PD-L1)-positive advanced solid tumors, including EC, who were treated with the anti-programmed cell death 1 (PD-1) monoclonal antibody pembrolizumab. Data from this trial found that higher response rates and longer PFS are significantly associated with higher T-cell-inflamed gene-expression profile (GEP), PD-L1 expression and tumor mutational burden (TMB; T-cell-inflamed GEP, P=0.012 and 0.017, n=203; PD-L1, P=0.018 and 0.005, n=198; TMB, P=0.018 and 0.051, n=77) (18). T-cell inflamed GEP was evaluated based on the normalized expression values of 18 selected genes using RNA extracted from FFPE tumor tissues (19). PD-L1 expression was evaluated by IHC, which was used to calculate the combined positive score [CPS; the number of PD-L1-positive cells (tumor cells, lymphocytes, macrophages) divided by the total number of viable tumor cells ×100]. TMB was assessed by whole-exome sequencing using DNA isolated from the FFPE tissues. This finding suggests that higher T-cell-inflamed GEP, PD-L1 expression and/or TMB may predict the efficacy of pembrolizumab in advanced EC.

The KEYNOTE-158 phase II trial assessed pembrolizumab monotherapy in previously treated, advanced but incurable solid tumors (n=790), including EC (n=82). TMB was evaluated in FFPE tumor tissues using the FoundationOne CDx assay (20). TMB-high was defined as ≥10 mutations per megabase. The response rates of TMB-high and TMB-low groups were found to be 29 vs. 6%, suggesting that TMB can be used to predict the efficacy of pembrolizumab in patients with previously treated, advanced EC.

The KEYNOTE-775 phase III trial assessed the efficacy of lenvatinib, a multikinase inhibitor of VEGFR1-3 and other receptor tyrosine kinases, combined with pembrolizumab or chemotherapy, in 827 patients with advanced EC who had previously received ≥ one platinum-based chemotherapy regimen (21). PFS was found to be longer in the lenvatinib plus pembrolizumab group compared with that in the lenvatinib plus chemotherapy group in both mismatch repair (MMR) proficient (HR, 0.60; 95% CI, 0.50–0.72; P<0.001) and in all patients (HR, 0.56; 95% CI, 0.47–0.66; P<0.001). In addition, OS was longer with in the lenvatinib plus pembrolizumab group compared with that in the lenvatinib plus chemotherapy group in both MMR proficient (HR, 0.68; 95% CI, 0.56–0.84; P<0.001) and all patients (HR, 0.62; 95% CI, 0.51–0.75; P<0.001). MMR status was determined by IHC staining of MLH1, MSH2, MSH6 and PMS2 proteins. These results suggest that lenvatinib plus pembrolizumab is efficacious for advanced EC irrespective of the MMR status. However, it should be noted that lenvatinib was discontinued due to drug-related adverse events in 22.7% of the patients, where the most frequent grade ≥3 adverse event was hypertension (37.9%) and was clinically significant for lenvatinib (21). Accordingly, considering the results of tumor assessment in terms of MSI/MMR status, T-cell-inflamed GEP and PD-L1 expression, coupled with using TMB for predicting pembrolizumab efficacy, may still be important even for this regimen in case of switching to pembrolizumab monotherapy.

### Cervical cancer

KEYNOTE-826 phase III trial assessed the efficacy of pembrolizumab compared with placebo in 617 patients with persistent, recurrent or metastatic cervical cancer who were also receiving platinum-based chemotherapy with or without bevacizumab. PFS (P<0.001) and OS (P<0.001) were found to be significantly longer with pembrolizumab compared with those in placebo (22). The HR (95% CI) for disease progression or death were 0.94 (0.52–1.70) for PD-L1 CPS <1, compared with 0.68 (0.49–0.94) for CPS 1 to <10 and 0.58 (0.44–0.77) for CPS ≥10. Likewise, the HR for death were 1.00 (0.53–1.89) for PD-L1 CPS <1, compared with 0.67 (0.46–0.97) for CPS 1 to <10 and 0.61 (0.44–0.84) for CPS ≥10. These findings suggest that PD-L1 expression can be used to predict the efficacy of adding concurrent pembrolizumab to chemotherapy in persistent, recurrent or metastatic cervical cancer.

## 4. Biomarkers for the early diagnosis/prevention of cancer

Biomarkers that can facilitate the early diagnosis or prevention of cancer are expected to enable the provision of an optimal cost-effective and ideal healthcare plan.

### Cervical cancer

High risk (HR)-human papilloma virus (HPV) DNA genotyping is more sensitive compared with cytology, rendering them useful for long-term risk prediction. By contrast, cytology has high specificity (apart from atypical squamous cells of undetermined significance) and is useful for estimating immediate risk, but has lower sensitivity and lower negative predictive value compared with HR-HPV DNA genotyping (23). HPV 16 or 18 infections have the highest risk of cervical intraepithelial neoplasia (CIN) 3 and occult cancer development, requiring colposcopy with targeted biopsy even when cytology results turn out negative (24).

Cyclin-dependent kinase inhibitor 2A (p16 INK4A) is a cyclin-dependent kinase inhibitor that can inhibit cyclin-dependent kinases 4 and 6, inducing G_1_ cell cycle arrest. Degradation of the tumor suppressor retinoblastoma (Rb) protein by the HR-HPV oncoprotein E7 and E2F upregulation result in a feedback loop, leading to the increased expression of p16 (25). p16 IHC staining has been reported to be 86.7% sensitive and 82.8% specific for ≥CIN 2 (CIN 2 or worse) diagnoses, rendering this useful for distinguishing high-grade CIN from ≤CIN 1 (26). When p16 staining is combined with H&E staining, the sensitivity for high-grade CIN is increased by 13%, decreasing the false-negative rate by 50% (27).

### Ovarian cancer

Hereditary breast and ovarian cancer (HBOC) is an autosomal dominant hereditary cancer predisposition syndrome that is caused by pathogenic germline *BRCA1/2* variants. The life-time risk for developing ovarian cancer in individuals harboring *BRCA1* mutations is 39–48%, compared with 11–20% in those harboring *BRCA2* mutations (28–31). To date, an effective screening method for improving the survival rate of ovarian cancer has remained elusive (32–36). Women with HBOC are recommended to receive risk-reducing salpingo-oophorectomy (RRSO), which has been shown to reduce mortality according to results from large-population prospective studies (37,38). Specifically, RRSO reduced mortality in individuals with *BRCA1* mutations aged 35–40 years and in individuals with *BRCA2* mutations aged 40–45 years. This appeared to be due to later ovarian cancer onset in carriers of *BRCA2* mutations compared with their *BRCA1* counterparts (37), after childbearing age (39). Serous tubal intra-epithelial carcinoma (STIC) is an early precursor for high-grade serous carcinoma of fallopian tube origin and is incidentally found in RRSO specimens (40–42). Coupling IHC results of p53 and Ki-67 with histological morphology has been found to improve the reproducibility of successfully pathologically diagnosing STIC (43). Ki-67 is a nuclear non-histone protein that is expressed during the G_1_, S and G_2_ phases, with peak expression at the M phase of the cell cycle but is typically absent at the G_0_ phase (44).

### EC

Lynch syndrome (LS) is an autosomal dominant hereditary cancer predisposition condition. It is diagnosed by the presence of germline pathogenic variants in one of the MMR genes *MLH1, MSH2, MSH6, PMS2* and epithelial cell adhesion molecule (45). LS is screened by MMR IHC and/or MSI testing on tumor tissues (46,47), specifically the loss of MLH1, MSH2, MSH6 and PMS2 expression (47). Detection of the loss MLH1 expression is followed by *MLH1* promoter methylation testing, where the presence of its hypermethylation would be deemed as a sporadic tumor instead of LS (47,48). MSI testing is conducted by comparing the PCR amplicons of microsatellite repeats in the tumor and those in the corresponding normal control. The life-time risks of developing colorectal cancer and EC in women with LS are 30–52 and 28–60%, respectively (49–53). However, although risk-reducing surgery for preventing EC in women with LS can reduce the incidence (54), it has not been reported to reduce mortality (55).

## 5. Molecular interpretations

**Figure 2.**
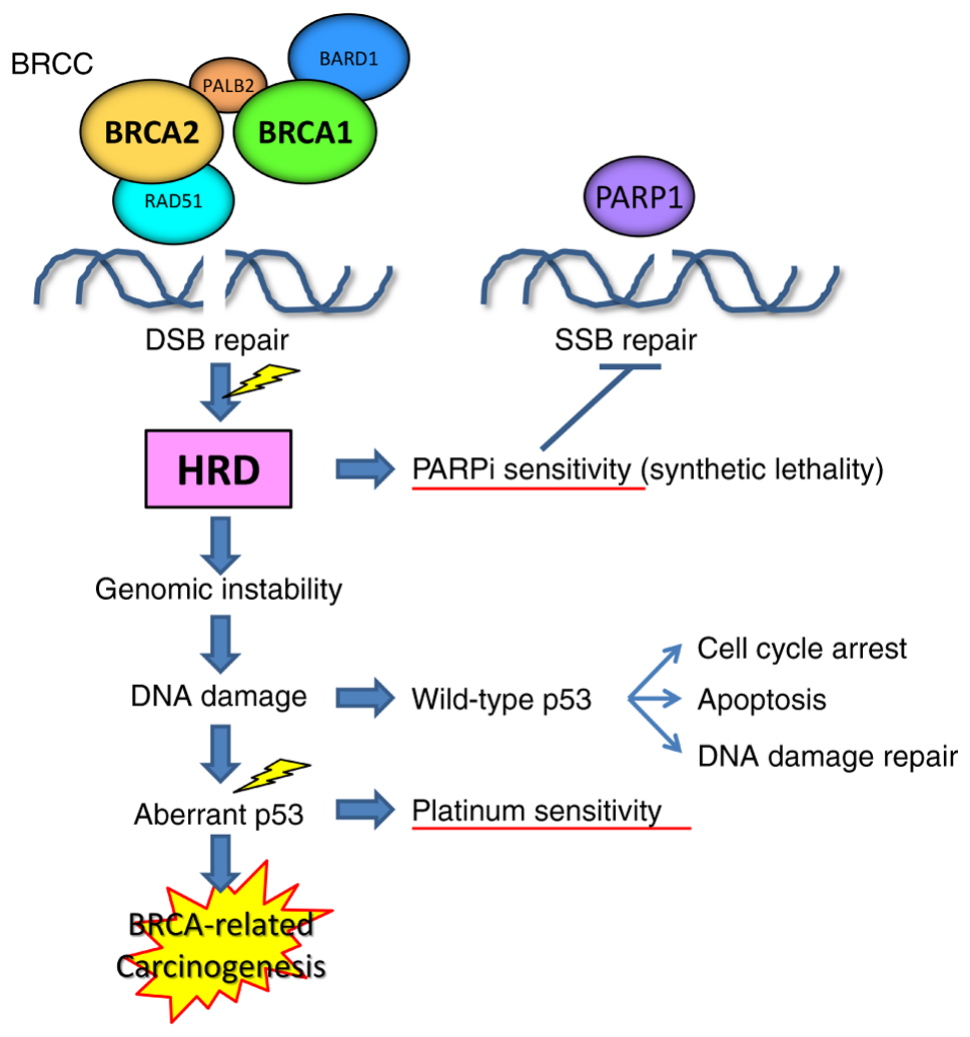
Proposed molecular mechanism of BRCA-related carcinogenesis. Tumors with loss of heterozygosity in *BRCA1* or *2* show HRD, which causes the accumulation of DNA damage, leading to cell cycle arrest and apoptosis induced by wild-type p53. Acquisition of p53 aberrations overcomes cell cycle arrest and circumvents apoptosis, resulting in BRCA-related carcinogenesis. BRCA, breast cancer gene; BRCC, BRCA1/BRCA2-containing complex; DSB, double-strand DNA break; SSB, single-strand DNA break; HRD, homologous recombination deficiency; PARPi, poly-(ADP ribose) polymerase inhibitor.

BRCA1, BRCA2, BRCA1-associated RING Domain 1, RAD51, BRCC36, BRCC45 *etc*. make up the BRCA1/BRCA2-containing complex (BRCC), which is involved in the homologous recombination-mediated repair of DNA double-strand breaks (Fig. 2) (56,57). Tumors with the loss of heterozygosity in either the *BRCA1* or *2* gene correspondingly show defects in repairing double-strand DNA breaks, and are sensitive to inhibitors of PARP1, an enzyme that contributes to repairing single-strand DNA breaks, by causing the synthetic lethality of tumor cells. Tumors with HRD tend to show genomic instability, accumulate DNA damage, undergo cell cycle arrest and apoptosis in a wild-type p53-dependent manner, which is pivotal for the DNA damage response (58–60). Acquisition of p53 aberrations, which appear to be an early and requisite event during BRCA-related carcinogenesis (61,62), overcomes cell cycle arrest and circumvents apoptosis, causing dysregulated proliferation (58–60). p53 dysfunction also causes defects in DNA damage repair, leading to sensitivity to DNA-damaging chemotherapeutics, such as platinum agents.

**Figure 3.**
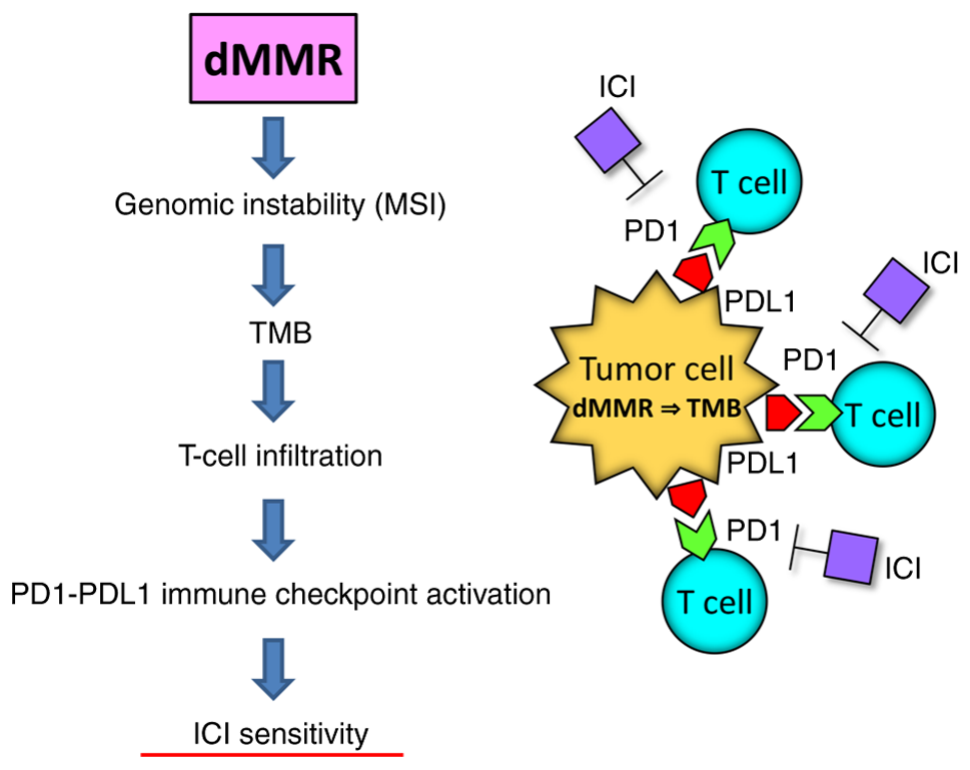
Mechanism underlying tumor sensitivity to ICI. Tumors with dMMR show high MSI and TMB, which causes T-cell infiltration and activates the PD1/PD-L1-mediated immune checkpoint pathway, consequently acquiring sensitivity to immune checkpoint blockade. dMMR, deficient mismatch repair; MSI, microsatellite instability; TMB, tumor mutation burden; ICI, immune checkpoint inhibitor; PD1, programmed cell death protein 1; PD-L1, programmed death-ligand 1.

Tumors with MMR deficiency show high MSI and TMB, which promotes the T-cell inflammatory phenotype and activation of the PD1/PD-L1-mediated immune checkpoint pathway (Fig. 3) (63). These tumors are sensitive to immune checkpoint inhibitors, such as anti-PD1 antibodies. Endometrial carcinomas with *POLE* mutations can be treated by surgery alone, leading to favorable prognoses. Surgery alone also avoids the need of overtreatment to maintain a good quality of life (QOL). By contrast, endometrial carcinomas with p53 aberrations tend to have the worst prognosis. They may be treated with conventional adjuvant therapies based on clinicopathological risk factors or pembrolizumab plus lenvatinib, combined with surgery.

**Figure 4.**
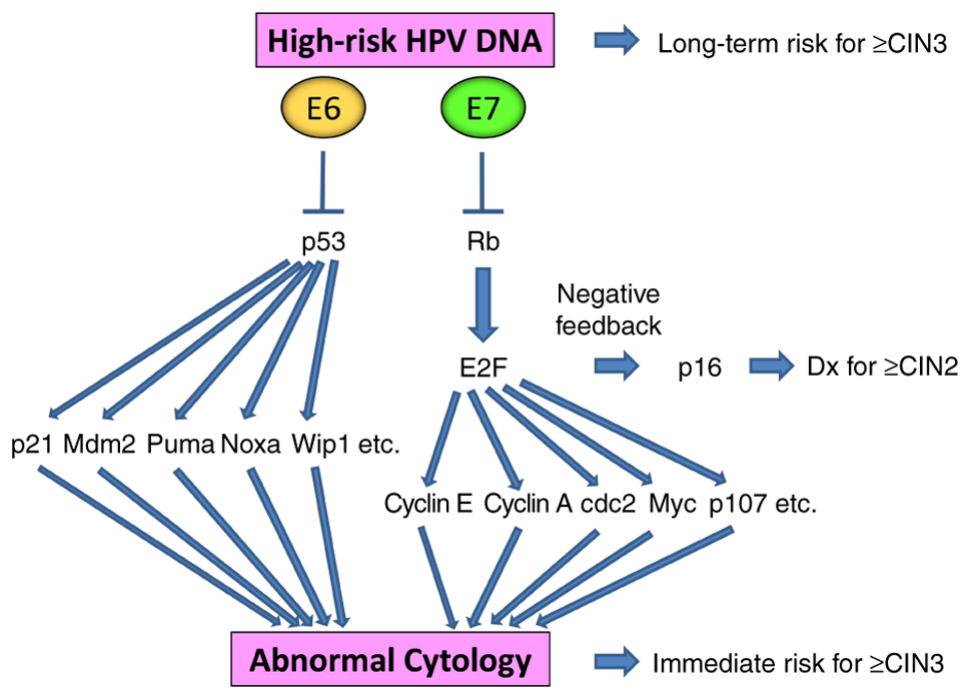
HPV-induced cervical carcinogenic pathways. Persistent infection with high-risk HPVs leads to the overexpression of E6 and E7, which degrade and inactivate p53 and Rb, respectively. This results in cervical carcinogenesis through multiple downstream pathways. The presence of high-risk HPV DNA corresponds to future long-term risk for developing ≥CIN3 (CIN3 or worse), whilst abnormal cytology corresponds to immediate risk of existing ≥CIN3. Degradation of Rb by E7 and upregulation of E2F result in a feedback loop, leading to the overexpression of p16, which supports the pathological diagnosis of ≥CIN2 (CIN2 or worse). HPV, human papillomavirus; Rb, retinoblastoma; CIN, cervical intraepithelial neoplasia; Dx, diagnosis.

Persistent infection with high-risk HPVs causes cervical carcinogenesis due to the chronic overexpression of viral oncoproteins E6 and E7, which degrade and inactivate tumor suppressors p53 and Rb, respectively. This in turn regulates a variety of cellular functions, such as apoptosis, cell cycle arrest, DNA damage response, immune system response, differentiation, transformation and immortalization. Therefore, whilst cytological changes are the results of cellular transformation, the presence of high-risk HPV DNA can reflect both the resultant status and future risk of transformation. Degradation of Rb by E7 and E2F upregulation results in a feedback loop, leading to p16 overexpression, which then supports the histological diagnosis for ≥CIN2 (Fig. 4).

## 6. Future perspectives

Clinical studies are currently ongoing to investigate the advantage of risk stratification using molecular biomarkers over conventional clinicopathological factors for the treatment of EC (Table I). PORTEC-4a phase III randomized trial is currently recruiting patients (9). It compares standard adjuvant vaginal brachytherapy with adjuvant treatment assignment (observation, vaginal brachytherapy or external beam radiotherapy) based on integrated clinicopathological and molecular risk profiles. It also performs the same evaluations as ProMisE for stage I–II EC (9). TAPER is a single-arm prospective cohort study that is also recruiting patients. It intends to investigate whether early-stage EC with *POLE* mutations or wild-type p53 carries a lower risk of pelvic recurrence at 3 years following no or de-escalated adjuvant therapy (10). The RAINBO trial (NCT05255653), which consists of four clinical trials investigating novel adjuvant therapies, is also recruiting patients (64). Patients are assigned to one of the following trials according to the molecular profile of their tumor: i) p53 abnormal to the p53abn-RED trial; ii) MMR deficient to the MMRd-GREEN trial; iii) no specific molecular profile to the NSMP-ORANGE trial; and iv) *POLE* mutant to the *POLE*mut-BLUE trial. The p53abn-RED randomized phase III trial compares adjuvant chemoradiation with/without 2 years of following treatment with olaparib. The MMRd-GREEN randomized phase III trial compares adjuvant pelvic external-beam radiotherapy with/without combined and following durvalumab, a human monoclonal anti-PD-L1 antibody, for 1 year. The NSPM-ORANGE randomized phase III trial compares adjuvant pelvic external-beam radiotherapy with/without 2-year following treatment with progestogens. The *POLE*mut-BLUE phase II single-arm trial evaluates the de-escalation of adjuvant therapy: No adjuvant therapy for stage I–II and no adjuvant therapy or adjuvant pelvic external-beam radiotherapy for stage III. The results of these studies are expected to provide useful evidence for formulating genome-based therapeutic strategies for EC. In terms of ovarian cancer, the most compelling evidence on the application of biomarkers is for HGSC. However, ovarian cancer is comprised of a variety of histological types. A comprehensive molecular classification system beyond pathological morphology needs to be constructed, in a manner that is applicable for risk stratification and therapeutic selection. Additionally, although the majority of ovarian cancers are diagnosed at advanced stages at present, an effective surveillance method for early detection remains elusive. Therefore, biomarkers for such utility are eagerly anticipated. For cervical cancer, considering the global effort for the prevalence of HPV vaccination, biomarkers for increased efficiency and economical screening instead of those for efficacious treatment will be needed in the near future.

## 7. Conclusion

The present review provided an overview for the current evidence on the use of cancer-related biomarkers for gynecological malignancies. Due to the recent acceleration in the advancements of human genomics and therapeutic developments, the knowledge and application of biomarkers are fast becoming essential for maximizing therapeutic efficacy and patient QOL whilst minimizing overtreatment and waste of limited resources. Several biomarkers have been suggested to be viable for guiding therapies, such as companion diagnostics based on the data from mainly observational studies (Table I). However, currently ongoing and future prospective interventional studies are warranted. They are expected to provide robust evidence on potentially effective and beneficial biomarkers that are applicable for the prevention, diagnosis and treatment of gynecological cancers, to further facilitate genome-directed precision medicine.

## Figures and Tables

**Table I.** Selected studies on biomarkers for patient prognosis and tumor response in gynecological oncology.

Biomarker	Target disease	Sample size	Study for biomarker	ClinicalTrials.gov identifier	Study phase	Published year	(Refs.)
MSI, *POLE*m, copy number	Stage I–IV EC	232	Observational			2013	(6)
dMMR, *POLE*m, p53	Stage I–IV EC	133, 139, 141	Observational			2015	(7)
dMMR, *POLE*m, p53	Stage I–IV EC	319	Observational			2017	(8)
dMMR, *POLE*m, p53	Stage I–II EC	recruiting	Interventional	NCT03469674 (POTEC-4a)	3		(9)
*POLE*m, p53	Stage I–II EC	recruiting	Interventional	NCT04705649 (TAPER)			(10)
dMMR, *POLE*m, p53	Stage I–III EC	recruiting	Interventional	NCT05255653 (RAINBO)	2, 3		(64)
p53	High-risk, stage IB-III EC	93	Observational	NCT00411138 (PORTEC-3)	3	2020	(17)
T-cell-inflamed GEP, PD-L1, TMB	PD-L1^+^ advanced solid tumors (including EC)	203, 198, 77	Observational	NCT02054806 (KEYNOTE-028)	1b	2019	(18)
TMB (FoundationOne CDx)	Advanced, incurable solid tumors (including EC)	790 (82)	Observational	NCT02628067 (KEYNOTE-158)	2	2020	(20)
p53	HGSC	281	Observational			2021	(11)
PFI	g*BRCA*m^+^ OC	38, 46	Observational			2010	(13)
HRD (myChoice CDx)	Stage III–IV OC	667	Observational	NCT02477644 (PAOLA-1)	3	2019	(14)
HRD (myChoice CDx)	Recurrent, platinum-sensitive HGSC	105	Observational	NCT02354586 (QUADRA)	2	2019	(15)
PD-L1	Persistent/recurrent/metastatic CC	617	Observational	NCT03635567 (KEYNOTE-826)	3	2021	(22)

Refs, references; MSI, microsatellite instability; *POLE*m, polymerase epsilon mutation; EC, endometrial cancer; dMMR, DNA mismatch repair deficiency; GEP, gene-expression profile; PD-L1, programmed cell death ligand 1; TMB, tumor mutation burden; CDx, companion diagnostics; HGSC, high-grade serous ovarian cancer; PFI, platinum-free interval; g*BRCA*m, germline *BRCA* mutation; OC, ovarian cancer; HRD, homologous recombination deficiency; CC, cervical cancer.

## Data Availability

Not applicable.
